# Editorial: Diet, Inflammation and Colorectal Cancer

**DOI:** 10.3389/fimmu.2019.02598

**Published:** 2019-11-07

**Authors:** Sandra Gessani, Fränzel J. Van Duijnhoven, Maria Jesus Moreno-Aliaga

**Affiliations:** ^1^Center for Gender-Specific Medicine, Istituto Superiore di Sanità, Rome, Italy; ^2^Division of Human Nutrition and Health, Wageningen University & Research, Wageningen, Netherlands; ^3^Department of Nutrition, Food Science and Physiology, Centre for Nutrition Research, University of Navarra, Pamplona, Spain; ^4^Navarra's Health Research Institute (IdiSNA), Pamplona, Spain; ^5^CIBERobn Physiopathology of Obesity and Nutrition, Carlos III Health Institute, Madrid, Spain

**Keywords:** diet, obesity, inflammation, colorectal cancer, dietary factors

Excess adiposity, a worldwide-growing pathological condition, is a major risk factor for colorectal cancer (CRC) as well as an important indicator of tumor survival, prognosis and recurrence (1). Obesity-associated low-grade chronic inflammation represents a key aspect in the connection between diet and cancer and dietary factors, together with high body fatness, are recognized as important determinants of CRC risk (World Cancer Research Fund/American Institute for Cancer Research 2017—Continuous update project report: diet, nutrition, physical activity and colorectal cancer—Available at: wcrf.org/colorectal-cancer-2017).

This Research Topic was designed to provide the reader with an overview of the impact of body weight and adiposity, as well as of specific food compounds on the inflammatory status in health and disease states, such as CRC. We collected original and review articles featuring the role of specific food compounds in the regulation of immune response and their potential therapeutic implications, the effect of polyunsaturated fatty acids (PUFA), oligosaccharides, polyphenols and body weight in the modulation of inflammation and long-term disease outcomes, highlighting the link between diet, inflammation, and CRC.

Adipose tissue (AT) inflammation plays a key role in obesity-associated morbidities, including CRC. Several studies have evidenced the importance of AT dysfunction during obesity in tumor metabolism and malignancies progression (2). However, the underlying biological processes are complex and not yet precisely defined. With the aim to unravel the crosstalk between dysfunctional AT in obesity and tumorigenesis, Del Cornò et al. use a RNA-sequencing approach to characterize the transcriptomic profile of visceral adipocytes from lean and obese subjects with or without CRC. The study reveals a complex relationship between altered visceral adiposity in obesity and cancer. Thus, the adipocytes transcriptome was differentially affected by obesity (pathways associated with inflammation) and CRC (TGF-β signaling and extracellular matrix remodeling), although some common alterations were also observed. Interestingly, the body mass index (BMI) of the subjects deeply affects the transcriptional program (cell adhesion, angiogenesis, and metabolism) of visceral adipocytes in CRC patients. Therefore, the obesity-induced changes in visceral adipocytes may promote colorectal tumor progression. ω3 PUFA have been shown to attenuate AT inflammation (3), and to have beneficial effects on CRC, including risk decrease and as adjuvant therapy (4). Here, Del Cornò et al. show that DHA, an ω3 PUFA, can regulate the expression of adipocyte genes involved in processes potentially relevant to carcinogenesis, and found a differential regulation between ω3 and ω6 PUFA in adipocytes from lean and obese subjects.

Increased inflammasome activity in AT has been proposed as an important mediator of obesity-induced inflammation and insulin resistance development, known mechanisms through which obesity promotes CRC risk and progression. In their mini review, Ahechu et al., describe the role of inflammasomes, in general and the NLRP3 inflammasome specifically, in carcinogenesis. In addition, they focus on the potential of the inflammasome to form new therapeutic strategies for the prevention and treatment of obesity-associated CRC development.

In the last years, evidence has been achieved that specific dietary patterns or categories of food compounds as well as body weight are associated with increased or decreased CRC risk. Natural products promoting human health have become a hot topic and their potential use in the management of neoplastic diseases has been largely explored (5). Among them, the pectin oligosaccharides (POS) are regarded as a novel class of functional foods with multiple health-promoting properties including antibacterial and immune modulating activity that can be beneficial for controlling CRC risk. In their review, Tan et al. provide a very informative picture of the role of POS in CRC prevention and progression emphasizing the capacity of these compounds to modulate signaling pathways involved in oxidative stress and inflammation. By reviewing the body of evidence present in the literature the authors discuss the dual role of POS in inhibiting/activating these pathways and highlight the need of linking, either already known or novel POS structures, with their function to improve their potential use in clinical practice.

Dietary polyphenols are plant secondary metabolites that have been widely studied because of their potential protective properties in chronic/degenerative diseases including obesity, metabolic syndrome and cancer (6, 7). In this Issue, Mileo et al. focused in reviewing current evidence from preclinical studies and clinical trials about the potential beneficial role of polyphenols in CRC. The authors describe how polyphenols modulate multiple immunomodulatory pathways, which can contribute to counteract the inflammatory tumor microenvironment. Moreover, there is growing evidence about the relationships between gut microbiota composition, gut inflammation and the risk of colon carcinogenesis (8). In this context, the article also highlights that the cross-talk between dietary polyphenols and gut microbiota might also account for their immunomodulatory properties and therapeutic potential in CRC. Finally, dietary polyphenols may not only represent a chemopreventive treatment, but also might be helpful as sensitizers to chemo/radiotherapies and their combination could, therefore, minimize toxicity and side effects of conventional oncological therapies.

In contrast to the overwhelming evidence that a higher body fatness is associated with an increased risk of CRC, the relation between BMI, as a measure of body fatness, and overall mortality in CRC patients is less clear. Shahjehan et al. explore the complex link between BMI and overall mortality in a retrospective study among 3,799 patients from the Mayo Clinic. Within their analyses, the authors investigate whether the association between body fatness and overall mortality is different at different stages of the disease. In addition, they evaluate whether a change in someone's body fatness is related to overall mortality in CRC patients. Since body fatness is modifiable, it may be an important factor to improve outcomes in CRC patients in the future.

Summarizing, this collection of articles further highlights the importance of obesity-associated AT inflammation in creating a more favorable situation for cancer establishment, pointing to obesity, body weight and dietary compounds as key factors influencing CRC risk. The articles in this topic have provided more specific information on important mechanisms linking diet and CRC risk. However, the underlying mechanism for diet, obesity, and survival in CRC patients is less clear and more research should focus on that in the future.

## Author Contributions

All authors listed have made a substantial, direct and intellectual contribution to the work, and approved it for publication.

### Conflict of Interest

The authors declare that the research was conducted in the absence of any commercial or financial relationships that could be construed as a potential conflict of interest.

## References

[1] Martinez-Useros J Garcia-Foncillas J. Obesity and colorectal cancer: molecular features of adipose tissue. J Transl Med. (2016) 14:21. 10.1186/s12967-016-0772-5 26801617 PMC4722674

[2] Gucalp A Iyengar NM Hudis CA Dannenberg AJ. Targeting obesity-related adipose tissue dysfunction to prevent cancer development and progression. Semin Oncol. (2016) 43:154–60. 10.1053/j.seminoncol.2015.09.012 26970134 PMC4789163

[3] Martinez-Fernandez L Laiglesia LM Huerta AE Martinez JA Moreno-Aliaga MJ. Omega-3 fatty acids and adipose tissue function in obesity and metabolic syndrome. Prostaglandins Other Lipid Mediat. (2015) 121(Pt A):24–41. 10.1016/j.prostaglandins.2015.07.003 26219838

[4] Volpato M Hull MA. Omega-3 polyunsaturated fatty acids as adjuvant therapy of colorectal cancer. Cancer Metastasis Rev. (2018) 37:545–55. 10.1007/s10555-018-9744-y 29971573 PMC6133177

[5] Bishayee A Sethi G. Bioactive natural products in cancer prevention and therapy: Progress and promise. Semin Cancer Biol. (2016) 40–1:1–3. 10.1016/j.semcancer.2016.08.006 27565447

[6] Afshari K Haddadi NS Haj-Mirzaian A Farzaei MH Rohani MM Akramian F. Natural flavonoids for the prevention of colon cancer: a comprehensive review of preclinical and clinical studies. J Cell Physiol. (2019) 234:21519–46. 10.1002/jcp.28777 31087338

[7] Durazzo A Lucarini M Souto EB Cicala C Caiazzo E Izzo AA. Polyphenols: a concise overview on the chemistry, occurrence, and human health. Phytother Res. (2019) 33:2221–43. 10.1002/ptr.6419 31359516

[8] Saus E Iraola-Guzman S Willis JR Brunet-Vega A Gabaldon T. Microbiome and colorectal cancer: roles in carcinogenesis and clinical potential. Mol Aspects Med. (2019) 69:93–106. 10.1016/j.mam.2019.05.001 31082399 PMC6856719

